# Supplementary material for: RELMα Is Induced in Airway Epithelial Cells by Oncostatin M without Requirement of STAT6 or IL-6 in Mouse Lungs In Vivo
Source: Cells. 2020 May 27;9(6):1338. doi: 10.3390/cells9061338 (PMC7349350; doi:10.3390/cells9061338)
Supplement: Supplementary file 1 [file cells-09-01338-s001.pdf]

Supplementary Figure 1

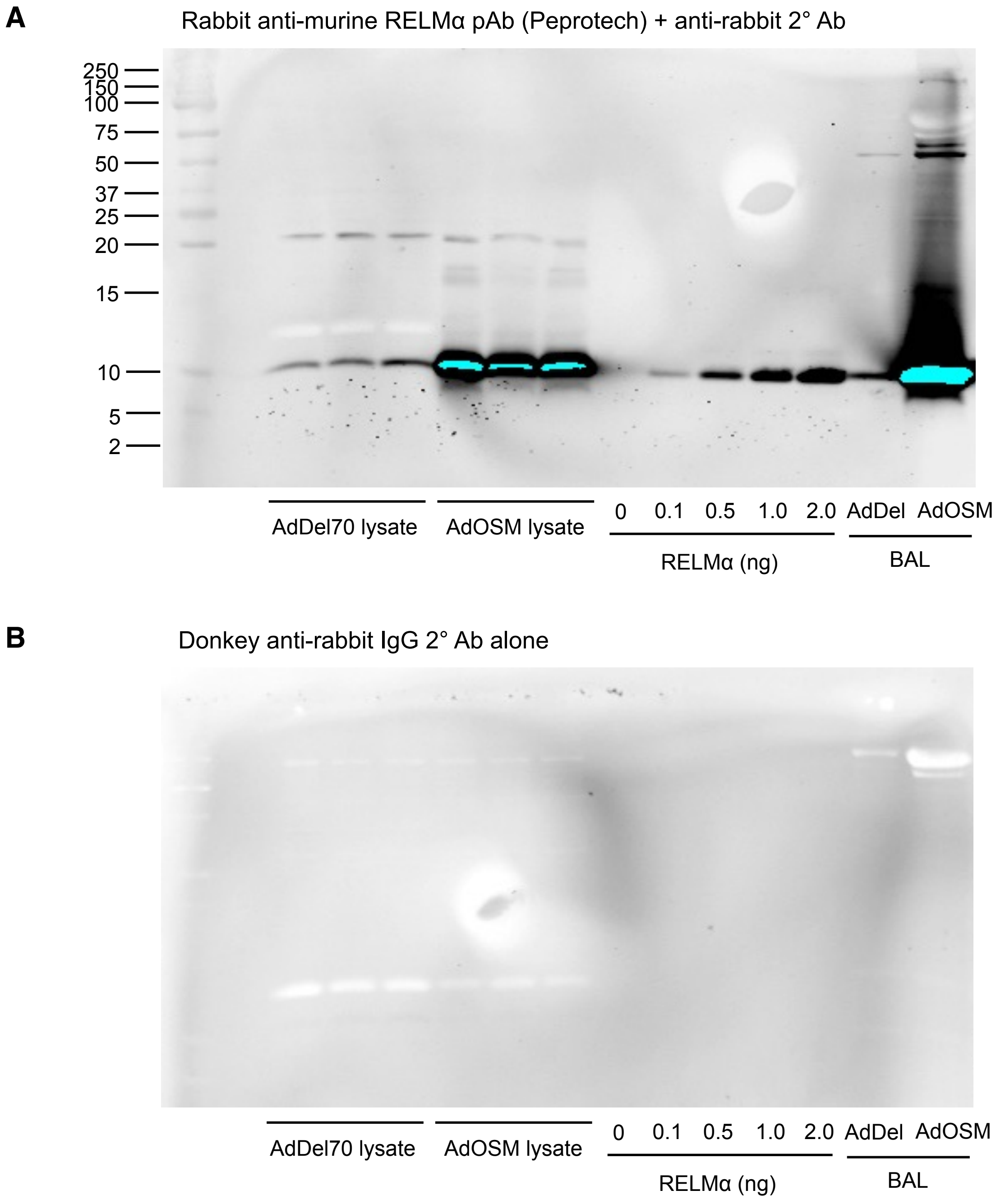

Supplementary Figure 1. Specificity of anti-murine RELMα polyclonal antibody by Western blot. Whole lung homogenates and BAL fluid from AdDel70- and AdOSM-treated C57Bl/6 mice were analyzed by Western blot. Known amounts of recombinant RELMα (0-2 ng) were also loaded on the same gel as a positive control and to generate a standard curve for semi-quantitative densitometric analyses. **(A)** Immunoblot was probed with rabbit anti-murine RELMα pAb and anti-rabbit secondary Ab. **(B)** Immunoblot was probed with anti-rabbit secondary Ab alone, showing that bands detected in **(A)** are not due to non-specific binding of the secondary Ab.

Supplementary Figure 2

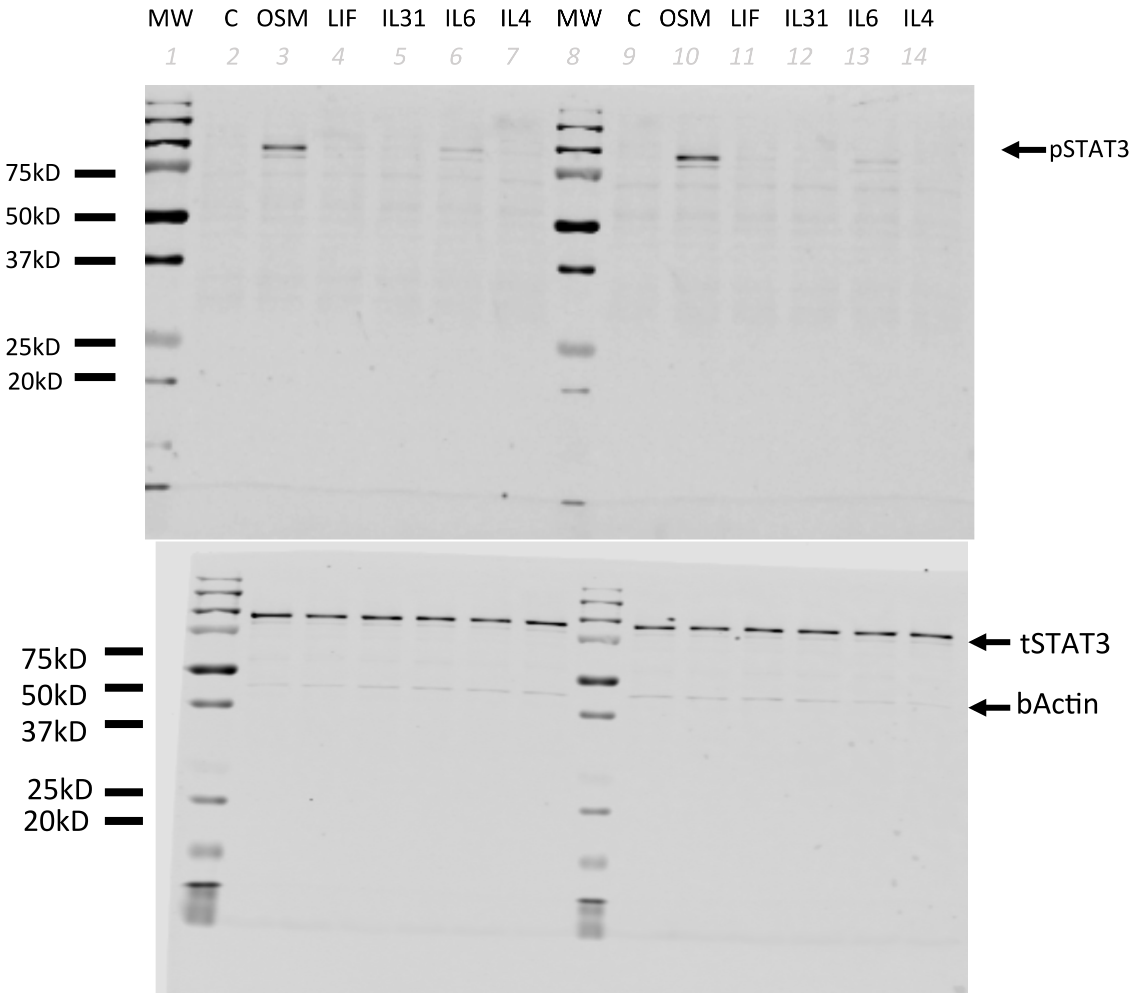

Supplementary figure 2: murine Tracheal-Bronchial Epithelial (TBE) cells stimulated with gp130-cytokines or IL-4. (Full blots, from fig 4)

C57Bl/6-derived murine TBE cells were stimulated for 1 hour with 20ng/ml of murine oncostatin M(OSM),leukemia inhibitory factor (LIF), interleukin-31 (IL-31), interleukin-6 (IL-6) or interleukin-4 (IL-4) and whole cell extracts probed for phospho-STAT3 (pSTAT3) by Western Blotting.

Supplementary figure 3

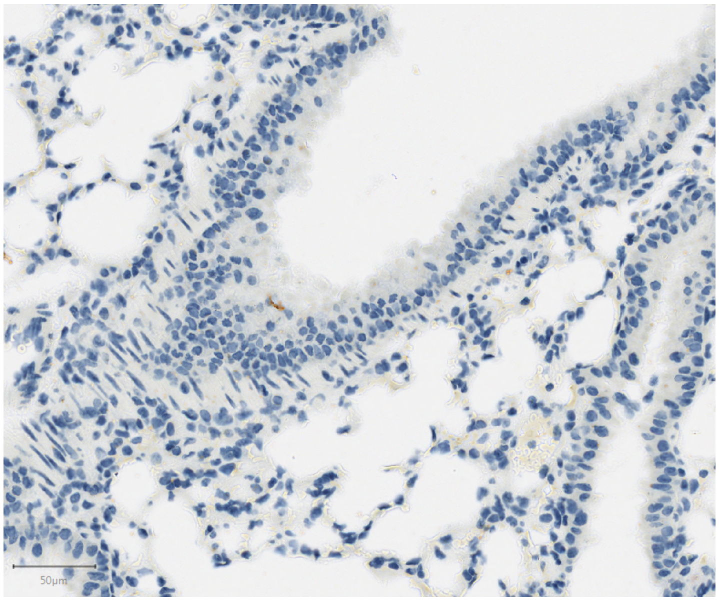

(A) AdOSM in IL-6-/- mice, No-probe control, From analysis in fig 4A

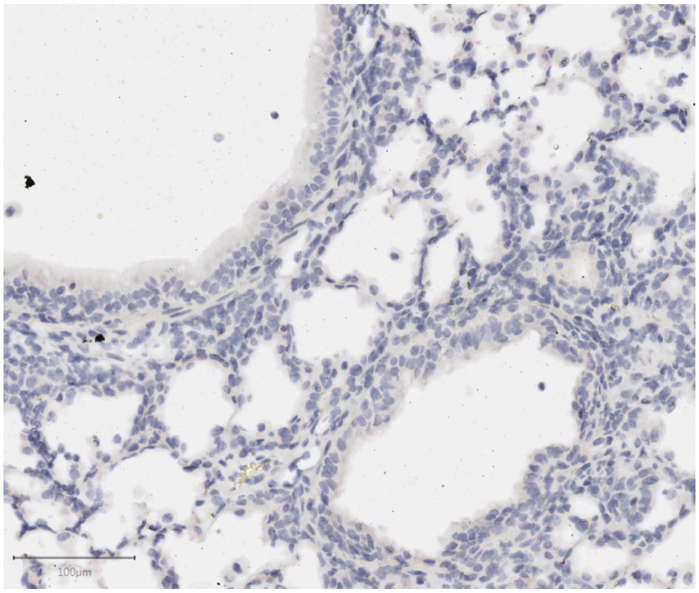

(B) AdOSM in STAT6-/- mice, No-probe control, From analysis in fig 4B

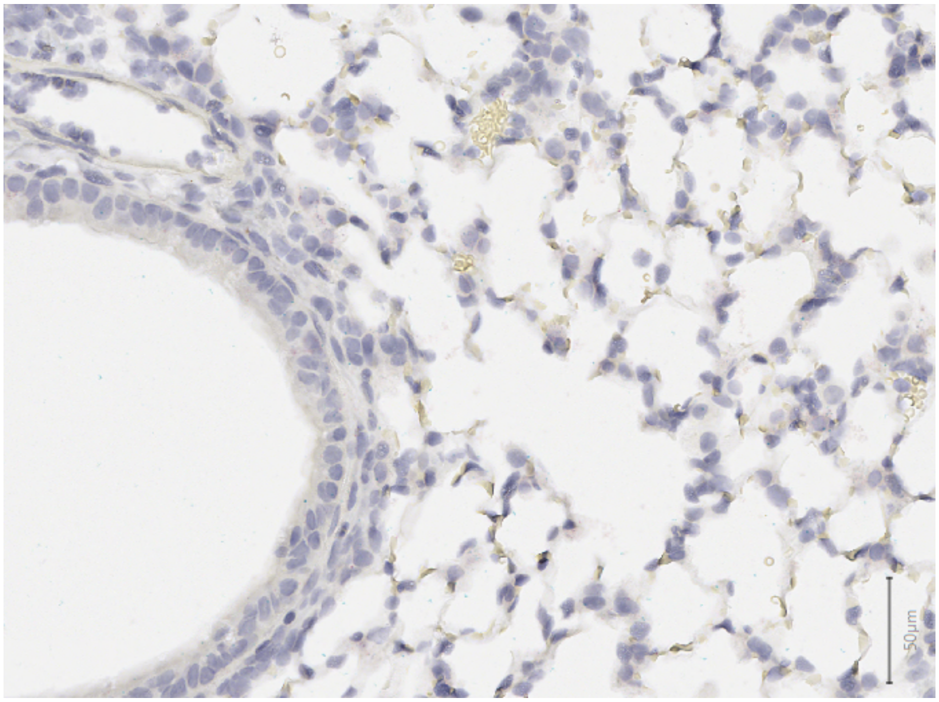

(C) AdOSM in wt mice, No-probe control (OSM red, YM-1 green), from analysis in fig 5B

Supplementary figure 3: Control stains (no-probe) in CISH procedure for fig 4 and 5. Representative section stains are shown for (A) AdOSM-treated IL-6-/- mice (control for Fig 4A in main figures), (B) Ad)SM treated STAT6-/- mice (control for Fig 4B in main figures) and (C) AdOSM treated wild type (wt) mice mice (control for Fig 5B in main figures). Methods included all CISH procedures except specific ACD probes:
